# Factors Affecting the Incidence, Progression, and Severity of COVID-19 in Type 1 Diabetes Mellitus

**DOI:** 10.1155/2021/1676914

**Published:** 2021-11-23

**Authors:** Amira S. Ahmed, Wejdan S. Alotaibi, Maha A. Aldubayan, Ahmad H. Alhowail, Amal H. Al-Najjar, Sridevi Chigurupati, Rehab M. Elgharabawy

**Affiliations:** ^1^Department of Pharmacology & Toxicology, College of Pharmacy, Qassim University, Qassim, Saudi Arabia; ^2^Hormones Department, Medical Research and Clinical Studies Institute, National Research Centre, Giza, Egypt; ^3^College of Pharmacy, Qassim University, Qassim, Saudi Arabia; ^4^Drug and Poison Information Specialist, Pharmacy Services, Security Forces Hospital, Riyadh, Saudi Arabia; ^5^Department of Medicinal Chemistry and Pharmacognosy, College of Pharmacy, Qassim University, Buraydah, Saudi Arabia; ^6^Department of Pharmacology & Toxicology, Faculty of Pharmacy, Tanta University, Tanta, Egypt

## Abstract

**Objectives:**

This study screened for factors affecting coronavirus disease 2019 (COVID-19) incidence in type 1 diabetes mellitus (T1DM) patients, appraised vitamin D's efficacy in preventing COVID-19, and assessed the effects of clinical characteristics, glycemic status, vitamin D, and hydroxychloroquine administration on COVID-19's progression and severity in T1DM patients.

**Methods:**

This retrospective research on 150 adults was conducted at Security Forces Hospital, Riyadh, KSA. Participants were allocated to three groups (50/group): control, T1DM, and T1DM with COVID-19. Participants' fasting blood glucose (FBG), glycated hemoglobin (HbA1c), complete blood count, vitamin D, C-reactive protein (CRP), erythrocyte sedimentation rate (ESR), ferritin, lactate dehydrogenase (LDH), prothrombin time, activated partial thromboplastin time, D-dimer, liver and kidney function, and hydroxychloroquine treatment were retrieved and analyzed.

**Results:**

The percentages of comorbidities and not taking hydroxychloroquine were significantly higher among T1DM patients with COVID-19 than patients with T1DM only. Mean vitamin D level was significantly lower in T1DM with COVID-19 patients than in the other two groups. Vitamin D showed a significant negative correlation with LDH, CRP, ESR, ferritin, and D-dimer, which was the most reliable predictor of COVID-19 severity in T1DM patients.

**Conclusion:**

Comorbidities and vitamin D deficiency are risk factors for COVID-19 in patients with T1DM. Patients who do not take hydroxychloroquine and have higher FBG and HbA1c levels are vulnerable to COVID-19. Vitamin D may be useful for preventing COVID-19 in T1DM patients. Comorbidities, higher FBG and HbA1c levels, not taking hydroxychloroquine, and vitamin D inadequacy elevate COVID-19 progression and severity in patients with T1DM.

## 1. Introduction

Type 1 diabetes mellitus (T1DM) is a mutual endocrine and metabolic condition that is widespread globally, with a continuously rising incidence of about 3% yearly [1, 2]. An autoimmune disease, T1DM, is triggered by insulin inadequacy due to pancreatic *β*-cell damage, causing hyperglycemia [3]. Eventually, T1DM patients are fully dependent on exogenous insulin, and vitamin D may be used to reduce the insulin dose. The immunomodulatory properties of vitamin D promote immune tolerance and the production of antibodies and decrease the inflammatory response. Patients with T1DM are at a high risk for serious complications, such as retinopathy, nephropathy, glycogen hepatopathy, and infection, especially respiratory infection [4].

In December 2019, serious cases of disease-inducing pneumonia and deaths were first reported in Wuhan, China. Soon after, the number of cases escalated and began to spread across the globe [5]. In January 2020, the World Health Organization announced that the coronavirus disease 2019 (COVID-19) outbreak was a public health emergency [6]. This new betacoronavirus (SARS-CoV-2) is related to severe acute respiratory syndrome coronavirus (SARS-CoV) and the Middle East respiratory syndrome coronavirus (MERS-CoV) because of their genetic similarities [7]. The risk factors for COVID-19 are diabetes, hypertension, and cardiovascular and respiratory diseases [8]. Diabetes is the most critical of the comorbidities, increasing the severity of COVID-19, which is attributed to the disruption of innate cellular immunity in T1DM patients [9, 10].

The pathogenesis of COVID-19 includes five phases: virus entry and spread, pathological findings, acute respiratory distress syndrome, cytokine storm, and finally, immune dysfunction [11]. Patients infected with the virus who require intensive care unit admission have high cytokine levels, indicating a link between the severity of the disease and cytokine storm [12]. Serum ferritin concentration is an indicator of the condition of iron stores in healthy individuals and of inflammatory diseases [13]. Cytokine storm induces vascular endothelial injury, causing hypercoagulability [14, 15]. Therefore, biomarkers, such as D-dimer (a biomarker of formation and degradation of fibrin), prothrombin time (PT), and activated partial thromboplastin time (APTT), are good indicators of infection severity [16].

Nearly half of the COVID-19 patients remain asymptomatic [17]. The COVID-19 infection could begin with flu like symptoms [18]. Patients with positive SARS-CoV-2 show lymphopenia and other manifestations, such as fatigue, fever, dry cough, anorexia, myalgia, dyspnea, diarrhea, nausea, dizziness, and abdominal pain [19]. The management of COVID-19 requires support measures based on each patient's clinical condition. Supportive treatments, including antibiotics and/or corticosteroids, are used when inflammatory biomarkers are increased [20]. Hashem and his colleagues [21] showed that hydroxychloroquine ameliorates the progression of the disease and can be used as a prophylaxis.

One of the main problems during the outbreak was the high numbers of patients admitted to hospitals. The admissions exceeded the available human and mechanical capacity, especially the capacity to provide critical care support. In these situations, risk stratification measures would be useful [22]. Therefore, early detection of accurate predictors of clinical outcomes are urgently needed [12]. Various hemogram parameters were studied in COVID-19 disease. These include neutrophil/lymphocyte count ratio and red cell distribution [23]. Additionally, D-dimer, C-reactive protein (CRP), and the erythrocyte sedimentation rate (ESR) can help identify a potentially poor prognosis [24], allowing the correct intervention to be implemented for each individual patient. The current study was conducted to screen for factors affecting the COVID-19 incidence in T1DM patients, to appraise the preventive efficacy of vitamin D against COVID-19 in T1DM patients and to assess the effects of clinical characteristics, vitamin D, and hydroxychloroquine administration on the COVID-19 progression and severity.

## 2. Patients and Methods

### 2.1. Patients

**Figure 1:**
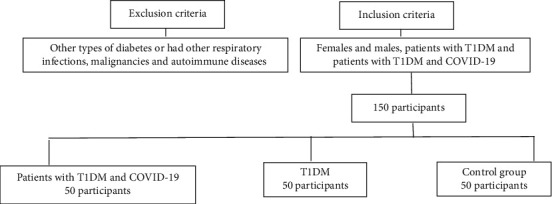
Enrollment of participants in the study. TIDM: type 1 diabetes mellitus.

This cross-sectional retrospective study was conducted with an adult population from Security Forces Hospital, Riyadh, Kingdom of Saudi Arabia. A total of 150 participants were recruited in accordance with study's inclusion and exclusion criteria. All participants were allocated to three groups of 50 subjects each: control group, patients with T1DM, and patients with T1DM and COVID-19 (Figure 1).

The ethics committee of the hospital approved the study. Data on patients' gender, age, current medications, duration of diabetes, diabetic complications, comorbidities, and hydroxychloroquine treatment were retrieved from medical records and recorded.

The types of patients treated and the treatments provided for COVID-19 included (a) patients with upper respiratory tract infections (with no O^2^ requirements or evidence of pneumonia), who received supportive care as needed; (b) patients with pneumonia (not intensive care unit (ICU) admissions) who received ceftriaxone +/- azithromycin, triple combination therapy (lopinavir/ritonavir, ribavirin and interferon beta-lb) and supportive care as needed; and (c) patients with pneumonia (not ICU admissions), who received piperacillin/tazobacatm +/- vancomycin if methicillin-resistant Staphylococcus aureus risk factors were identified, triple combination therapy (lopinavir/ritonavir, ribavirin, and interferon beta-lb) or remdesivir, if available, and supportive care as needed.

### 2.2. Inclusion and Exclusion Criteria

The patients who were randomized included in the study consisted of females and males, patients with T1DM, and patients with T1DM and COVID-19. Patients were excluded from the study if they were diagnosed with other types of diabetes or had other respiratory infections, malignancies, and autoimmune diseases.

### 2.3. Data Collection

Data were retrieved from the medical records of potential participants in 2020. Fasting blood glucose (FBG, human diagnostica liquicolor test, Germany); glycated hemoglobin (HbA1c, BioSystems, Spain); complete blood count (CBC, Sysmex hematology analyzer, Japan); vitamin D (Fisher Scientific International, Inc., New York); inflammatory markers including CRP (R and D Systems, Inc., USA), ESR (Fisher Scientific International, Inc., New York), and ferritin (Roche Diagnostics Elecsys and Cobas, Schweiz); lactate dehydrogenase (LDH, USCN Business Co., Ltd, Wuhan); PT, APTT (BioSystems, Spain); D-dimer (USCN Business Co., Ltd, Wuhan); and liver and kidney function tests were recorded for all participants.

### 2.4. Statistical Analyses

The statistical package SPSS, version 21, (IBM, Armonk, USA) was used to analyze the data. A sample size of 46 patients was required in each group to detect an effect size of 0.65 between each pair of groups with an 80% power using a 5% level of significance with *p* value correction using the Bonferroni adjustment. Thus, 50 patients were recruited in each group. The Kolmogorov-Smirnov test was used to analyze the distribution of variables. The data were represented as mean ± standard deviation (SD) and number (%). Tukey-Kramer post hoc comparisons were performed after a significant result was found in a one-way analysis of variance (ANOVA). Pearson's chi square test was used to examine differences in the proportions of the categorical variables and evaluate the associations between distinct variables. Pearson's correlation (*r*) was applied to assess the associations of the distinct parameters with one another. The receiving operating characteristic curve was plotted with the sensitivity values plotted versus 1-specificity. The accuracy of the biochemical markers' predictions of COVID-19 severity in T1DM patients was the sensitivity and specificity average. The statistical significance level in this study was established to *p* < 0.05.

## 3. Results

### 3.1. Demographic and Clinical Characteristics of the Participants

No significant difference in age or gender was found among the three study groups. Neither was a significant difference in the duration nor complications of T1DM found among the three groups. The percentage of comorbidities, such as hypertension, hyperlipidemia, asthma, and iron deficiency anemia in patients with T1DM and COVID-19 (61.2%), was significantly higher than that in the patients with T1DM only (36.7%) (*χ*^2^: 5.880, *p* = 0.015). None of the T1DM patients with COVID-19 had taken hydroxychloroquine previously; thus, the percentage of the patients in this group treated with hydroxychloroquine was significantly lower compared to the percentage of T1DM patients without COVID-19 (*χ*^2^: 22.609, *p* < 0.001) as presented in Table 1.

### 3.2. Diabetic Biomarkers and Vitamin D Levels of the Participants

Diabetic biomarkers and vitamin D levels were significantly different among the three groups (*p* < 0.001; see Table 2). The FBG and HbA1c levels were significantly greater in both the T1DM patients with and without COVID-19, compared to the control group. In patients with T1DM and COVID-19, the levels of diabetic biomarkers were higher than those of the patients with T1DM only. The mean vitamin D level was significantly lower in patients with T1DM and COVID-19 relative to the other two groups. Furthermore, the levels of vitamin D were significantly lower in the patients with T1DM compared to the control group (as seen in Table 2).

### 3.3. Complete Blood Count Profiles of the Participants

Table 3 shows the complete blood count profiles of the participants. No significant difference was found in the RBCs among the three groups; however, significant differences in the WBCs, lymphocytes, and platelets were found among the three study groups (*p* < 0.001). The mean WBC was significantly lower in the control group compared to the other two groups, and it was significantly elevated in patients with T1DM and COVID-19 compared to the patients with T1DM only. The mean lymphocyte and platelet levels were significantly lower in the T1DM patients infected with SARS-CoV-2 compared to the other two study groups. The platelet level of the patients with T1DM was significantly higher than that of the control group.

### 3.4. Inflammatory Biomarkers of the Participants

Table 4 shows that the CRP, ESR, and ferritin levels were significantly different among the three study groups (*p* < 0.001). The mean levels of all three inflammatory biomarkers were significantly higher in the T1DM patients with COVID-19 compared to the other two groups, and significantly higher levels were found in the T1DM patients without COVID-19 compared to the control group.

### 3.5. Coagulation Profile of the Participants

Table 5 presents the coagulation profile of the participants. The levels of all the coagulation biomarkers were significantly different among the three groups (*p* < 0.001). In the T1DM with COVID-19 patients, the mean PT and D-dimer levels were significantly higher than the levels in the other two groups. The mean APTT level was significantly higher in the T1DM patients with COVID-19 compared to the T1DM patients without COVID-19. The D-dimer level was significantly higher in the T1DM patients compared to the control group; however, the level of APTT was significantly lower in the T1DM patients without COVID-19, compared to the control group.

### 3.6. Participants' Test Results for Lactate Dehydrogenase and Liver and Kidney Function

Table 6 presents the results of participants' LDH and liver and kidney function tests. The LDH, alanine aminotransferase (ALT), aspartate aminotransferase (AST), and creatinine levels were significantly different among the three study groups (*p* < 0.001). The mean LDH, ALT, AST, and creatinine levels were significantly higher in the T1DM patients with and without COVID-19 compared to the control group. The mean LDH and creatinine levels were significantly higher in T1DM patients with COVID-19 than in the T1DM patients without COVID-19.

### 3.7. Correlations of the Clinical Characteristics, Diabetes Biomarkers, and Vitamin D Levels with Lactate Dehydrogenase and Inflammatory and Coagulation Biomarkers in Patients with T1DM with and without COVID-19

**Figure 2:**
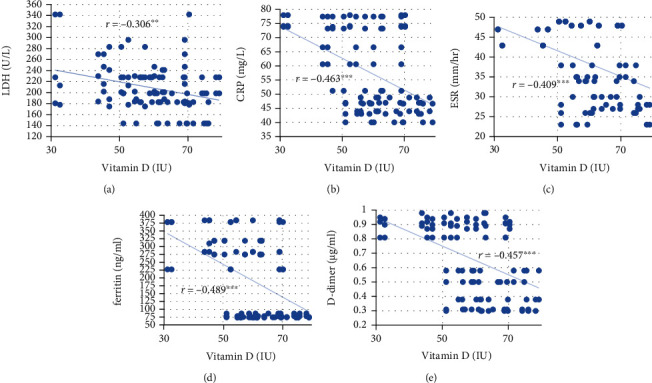
Correlations of vitamin D with (a) LDH, (b) CRP, (c) ESR, (d) ferritin, and (e) D-dimer in T1DM patients with and without COVID-19. Scatter plots show the levels of biomarkers at the levels of vitamin D. LDH: lactate dehydrogenase; CRP: C-reactive protein; ESR: erythrocyte sedimentation rate; T1DM: type 1 diabetes mellitus. ^∗∗^*p* < 0.01, ^∗∗∗^*p* > 0.001.

As presented in Table 7, comorbidities showed a significant positive correlation with CRP, ESR, and D-dimer (*p* < 0.05). A significant negative correlation of hydroxychloroquine administration with LDH (*p* < 0.05), CRP, ESR, ferritin, and D-dimer (*p* < 0.001) was found. A significant positive correlation was found between participants' FBG and LDH levels (*p* < 0.05), and their FBG and HbA1c levels showed a significant positive association with their CRP, ESR, ferritin, and D-dimer levels (*p* < 0.001). Vitamin D presented a significant negative correlation with participants' LDH (*p* < 0.01), CRP, ESR, ferritin, and D-dimer levels (*p* < 0.001) as presented in Figure 2.

### 3.8. Receiver-Operating Characteristic Curve of the D-Dimer, Prothrombin Time, and Partial Thromboplastin Time

**Figure 3:**
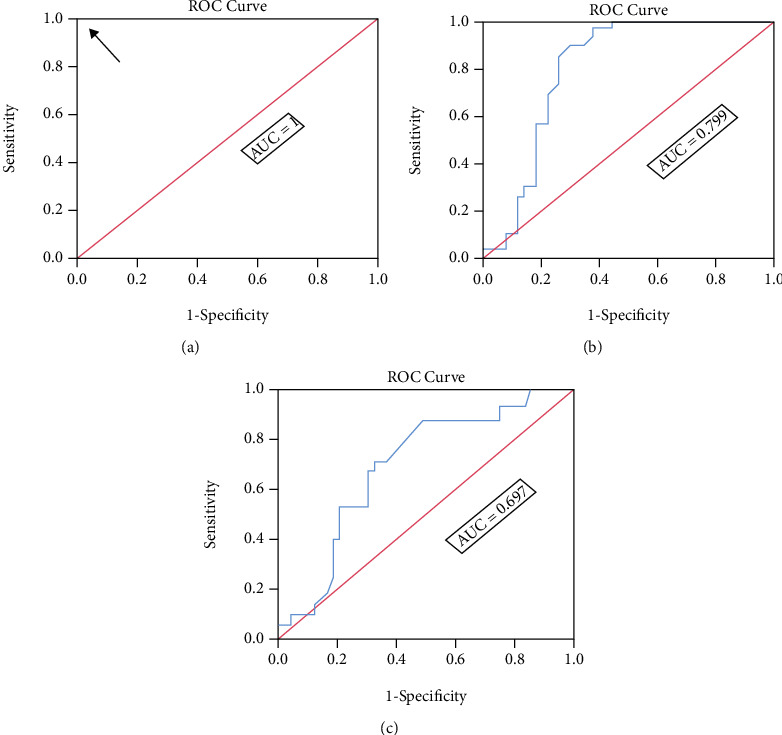
Area under the ROC curve for (a) D-dimer, (b) PT, and (c) APTT. AUC: area under the curve; ROC: receiver operating characteristic curve; PT: prothrombin time; APTT: activated partial thromboplastin time. The arrow refers to the best cut-off value.

As illustrated in Figure 3, the area under the curve for the D-dimer, PT, and APTT were 1, 0.799, and 0.697, respectively. Therefore, D-dimer was the most reliable predictor of the severity of COVID-19 in T1DM patients. The best cut-off value for D-dimer was 0.695 *μ*g/ml.

## 4. Discussion

The COVID-19 is a common health emergency. Approximately 20-50% of COVID-19 cases worldwide are diabetic patients [25]. In the present study, the comorbidities percentage in T1DM with COVID-19 patients (61.2%) was significantly greater than that in T1DM patients only (36.7%). The T1DM patients infected with SARS-CoV-2 had comorbidities, like hypertension, dyslipidemia, asthma, and iron deficiency anemia. Hypertension is associated with increased mortality from COVID-19 [26] and hypertensive patients are thought to have elevated expression of angiotensin-converting enzyme 2 (ACE2) related to genetic polymorphism. The use of angiotensin-converting enzyme inhibitors (ACEIs) or angiotensin-receptor blockers (ARBs) may increase the risk for or the severity of COVID-19 [27]. Treatment with ACEIs and ARBs upregulates ACE2 receptors in the lungs for the anchoring of spike (S) proteins on the exterior surfaces of SARS-CoV-2 to these receptors in the lower respiratory tract of infected patients in order to enter their lungs [28]. Evidence from studies shows that asthmatic patients are overrepresented among adults hospitalized with COVID-19, which is attributed to asthma exacerbation by SARS-CoV-2; thus, asthma is a risk factor for COVID-19 [1, 2].

Anemia might be related with the poor outcomes of COVID-19, causing hypoxia and multiple organ dysfunction, especially respiratory dysfunction. Additionally, the anemia severity in COVID-19 patients might worsen, which is caused by the interaction of SARS-CoV-2 with hemoglobin molecules on the erythrocyte through ACE2 and the CD26 and CD147 receptors. This viral-hemoglobin interaction causes the virus to attack the heme on the 1-*β* chain of hemoglobin, resulting in hemolysis. The SARS-CoV-2 may mimic the hepcidin action that increases circulating and tissue ferritin, while inducing serum iron deficiency and lack of hemoglobin. A ferritin increase will lead to ferroptosis with high lipid peroxidation and oxidative stress that can precipitate an inflammatory/immune overresponse (cytokine storm), resulting in a worse COVID-19 outcome [29].

The diabetes biomarker level in T1DM patients with COVID-19 was higher than that in the T1DM patients only. Ling et al. [30] and Wang et al. [31] found that increased levels of FBG and Hb1Ac are associated with the progression of COVID-19 in diabetic patients. Hyperglycemia prevents neutrophil chemotaxis, lowers phagocytosis by macrophages, neutrophils, and monocytes, and impairs innate cell-mediated immunity. Disrupted immunity in hyperglycemic patients permits the unhindered spread of pathogens within the host [32]. The current data are consistent with the results of a study by Cai and his colleagues [33], who observed a higher level of FBG in COVID-19 patients with diabetes, confirming the hyperglycemia role in COVID-19 development, progression, and severity.

In the current study, the mean vitamin D level in T1DM patients with COVID-19 was significantly lower compared to the levels in the other two study groups. Insufficient vitamin D affects immune function because of its immunomodulatory properties, [34], and it increases innate immunity by antiviral peptides secretion [35], which improves mucosal defenses against acute respiratory infections. The current results are consistent with a study by Ali [36] that concluded that vitamin D plays a crucial role in preventing SARS-CoV-2 infection and decreases the chances of the infection worsening, thereby reducing the severity and progression of the disease. Vitamin D deficiency is an easily adjusted risk factor for acute respiratory infections and should be actively managed by low-cost, safe, and easily obtainable vitamin D supplements [37].

In this study, none of the T1DM with COVID-19 patients had taken hydroxychloroquine previously; thus, a significantly lower patients' percentage in this group was treated with hydroxychloroquine, compared to the percentage of T1DM patients without COVID-19. Principi and Esposito [38] suggested the hydroxychloroquine use as a prophylactic measure that might help reduce pandemic's spread in countries with a high COVID-19 incidence. Hydroxychloroquine inhibits the coronavirus in several phases. The drug alters the pH at the cell membrane surface, preventing the fusion of the virus to the cell membrane. It suppresses nucleic acid replication, viral protein glycosylation, virus assembly, new virus particle transport, and virus release to achieve antiviral effects [39].

The present work comes in agreement with the study of Khalid et al. [40] who stated that ferritin, D-Dimer, CRP, LDH, and APTT levels in COVID-19 patients showed a statistically significant difference compared with standard values. The mean LDH level was significantly higher in T1DM with COVID-19 patients than in patients with T1DM only. The LDH level is widely used to diagnose tissue damage associated with a wide variety of conditions, including interstitial lung disease, suggesting viral infection or lung damage, such as pneumonia-induced SARS-CoV-2 [41]. Yan et al. [42] confirmed the correlation between LDH and mortality rate of COVID-19 patients.

In the present study, the mean ferritin level was significantly higher in patients with T1DM and COVID-19 compared to the other two study groups. Ferritin was also significantly elevated in patients with T1DM compared to the control group. This study's results are consistent with those of Cheng et al. [43], which found an association between a high level of ferritin with severe symptoms and a poor prognosis of the disease. Circulation of ferritin increases during viral infections; therefore, it is used as a biomarker for infections [44]. Increased ferritin levels from cytokine storm and secondary hemophagocytic lymphohistiocytosis have been recorded in extremely ill COVID-19 patients [45]. During a cytokine storm in COVID-19, several inflammatory cytokines are quickly formed, including interleukin 6, tumor necrosis alpha, interleukin 1*β*, interleukin 12, and interferon gamma, stimulating the secretion of ferritin by hepatocytes, Kupffer cells, and macrophages [46].

Our data revealed that the average level of D-dimer was significantly higher in the T1DM with COVID-19 patients than the other two study groups. The D-dimer level was significantly higher in the T1DM patients compared to the control group. Furthermore, D-dimer was found to be the most reliable predictor of severe COVID-19 in T1DM patients (with an area under the curve of 1). D-dimer elevation has been described to be one of the most significant common laboratory findings in COVID-19 patients in need of hospital admission [47]. Effective and early clinical outcome predictors are urgently needed for risk stratification of patients with COVID-19. D-dimer is derived from the development of cross-linked fibrin and represents the coagulation and fibrinolysis activation [48]. COVID-19 has been linked with hemostatic abnormalities, and significantly increased levels of D-dimer have been observed in nonsurvivors [49]. Connors and Levy [50] found that other coagulation biomarkers, such as PT and APTT, were elevated in COVID-19 patients, which is consistent with the current findings; therefore, anticoagulant therapy for critically ill patients was suggested.

The current study's results confirmed a significant positive correlation of FBG and HbA1c with LDH, inflammatory markers (CRP, ESR, and ferritin), and D-dimer. Furthermore, a significant positive correlation of comorbidities with CRP, ESR, and D-dimer was found, but vitamin D and hydroxychloroquine administration showed a significant negative correlation with LDH, CRP, ESR, ferritin, and D-dimer, increasing the COVID-19 progression and severity in T1DM patients.

Collection of data from only one hospital and undiagnosed conditions that could affect biomarkers' changes are considered limitation of the present study.

## 5. Conclusion

Comorbidities and vitamin D deficiency may be risk factors in the incidence of COVID-19 in T1DM patients. Patients with T1DM who do not take hydroxychloroquine and have higher FBG and HbA1c levels are vulnerable to SARS-COV-2 infection. Thus, vitamin D might be useful for preventing COVID-19 in T1DM patients. Finally, comorbidities, higher levels of FBG and HbA1c, not taking hydroxychloroquine, and vitamin D deficiency elevate the COVID-19 progression and severity in T1DM patients.

## Figures and Tables

**Table 1:** Demographic and clinical characteristics of the participants (*N* = 150).

Parameters	Groups				
	Control group	Type 1 diabetic patients	Type 1 diabetic patients with COVID-19	Test value	*p* value
*N*	50	50	50	—	—
Age (years)	36.08 ± 4.46	36.18 ± 3.52	36.71 ± 6.63	0.222	0.801
Gender					
Male	15 (30.6%)	24 (49%)	26 (51%)	5.037	0.081
Female	35 (69.4%)	26 (51%)	24 (49%)		
T1DM duration (years)	NA	14.53 ± .96	14.75 ± 3.03	0.043	0.672
Diabetic complications					
Yes	NA	20 (40.8%)	21 (42.9%)	0.042	0.838
None		30 (59.2%)	29 (57.1%)		
Comorbidities^#^					
Yes	NA	18 (36.7%)	31 (61.2%)	5.880	0.015
None		32 (63.3%)	19 (38.8%)		
Taking hydroxychloroquine					
Yes	NA	13 (26.5%)	0 (0%)	22.609	<0.001
No	NA	37 (73.5%)	50 (100%)		

Results are summarized as mean ± SD and *N* (%). ^#^Comorbidities, like hypertension, dyslipidemia, asthma, and iron deficiency anemia. *N*: number of participants; TIDM: type 1 diabetes mellitus; NA: not applicable. *p* < 0.05 is considered significant.

**Table 2:** Diabetic biomarkers and vitamin D level of the participants (*N* = 150).

Parameters	Groups				
	Control group	Type 1 diabetic patients	Type 1 diabetic patients with COVID-19	Test value	*p* value
FBG (mmol/l)	5.28 ± 0.72	15.81 ± 2.51^a^	19.12 ± 2.42^ab^	606.087	<0.001
HbA1c (%)	5.69 ± 0.39	9.4 ± 1.02^a^	10.8 ± 0.97^ab^	476.234	<0.001
Vitamin D (IU)	97.23 ± 20.35	64.98 ± 8.44^a^	53.93 ± 11.91^ab^	118.585	<0.001

Results are expressed as mean ± SD. FBG: fasting blood glucose; HbA1c: glycated hemoglobin. ^a^Significantly different from the control group. ^b^Significantly different from the type 1 diabetic patients. *p* < 0.05 is considered significant.

**Table 3:** Complete blood count profiles of the participants (*N* = 150).

Parameters	Groups				
	Control group	Type 1 diabetic patients	Type 1 diabetic patients with COVID-19	Test value	*p* value
RBCs (×1012 cells/l)	4.59 ± 0.77	4.54 ± 0.4	4.27 ± 0.84^b^	3.006	0.053
WBCs (×109 cells/l)	6.14 ± 0.71	7.27 ± 0.91^a^	9.68 ± 1.23^ab^	169.201	<0.001
LYM (%)	29.32 ± 6.25	29.13 ± 5.65	22.27 ± 3.68^ab^	28.063	<0.001
PLT (×109 cells/l)	261.04 ± 41.53	288.2 ± 62.33^a^	233.88 ± 42.1^ab^	14.694	<0.001

Results are expressed as mean ± SD. RBCs: red blood cells; WBCs: white blood cells; LYM: lymphocytes; PLT: platelets. ^a^Significantly different from the control group. ^b^Significantly different from the type 1 diabetic patients. *p* < 0.05 is considered significant.

**Table 4:** Inflammatory biomarkers of the participants (*N* = 150).

Parameters	Groups				
	Control group	Type 1 diabetic patients	Type 1 diabetic patients with COVID-19	Test value	*p* value
CRP (mg/l)	2.36 ± 0.49	44.89 ± 2.93^a^	69.87 ± 8.91^ab^	1940.289	<0.001
ESR (mm/hr)	16.59 ± 3.34	30.12 ± 4.78^a^	46.98 ± 2.14^ab^	883.013	<0.001
Ferritin (ng/ml)	44.33 ± 8.83	78.06 ± 4.77^a^	311.49 ± 56.17^ab^	955.780	<0.001

Results are expressed as mean ± SD. CRP: C-reactive protein; ESR: erythrocyte sedimentation rate. ^a^Significantly different from the control group. ^b^Significantly different from the type 1 diabetic patients. *p* < 0.05 is considered significant.

**Table 5:** Coagulation profile of the participants (*N* = 150).

Parameters	Groups				
	Control group	Type 1 diabetic patients	Type 1 diabetic patients with COVID-19	Test value	*p* value
PT (seconds)	11.47 ± 0.87	11.21 ± 1.91	13.13 ± 2.28^ab^	16.540	<0.001
APTT (seconds)	31.29 ± 3.48	29.55 ± 4.4^a^	32.16 ± 3.98^b^	5.508	<0.001
D-dimer (*μ*g/ml)	0.28 ± 0.08	0.42 ± 0.11^a^	0.9 ± 0.05^ab^	721.364	<0.001

Results are expressed as mean ± SD. PT: prothrombin time; APPT: activated partial thromboplastin time. ^a^Significantly different from the control group. ^b^Significantly different from the type 1 diabetic patients. *p* < 0.05 is considered significant.

**Table 6:** Lactate dehydrogenase level and liver and kidney function tests of the participants (*N* = 150).

Parameters	Groups				
	Control group	Type 1 diabetic patients	Type 1 diabetic patients with COVID-19	Test value	*p* value
LDH (U/l)	157.5 ± 9.6	188.5 ± 30.34^a^	229 ± 45.15^ab^	61.935	<0.001
ALT (U/l)	13.33 ± 2.25	25.82 ± 4.97^a^	26.31 ± 4.82^a^	149.762	<0.001
AST (U/l)	15.31 ± 2.27	27.78 ± 5.5^a^	27.82 ± 3.91^a^	150.723	<0.001
Creatinine (mg/dl)	52.35 ± 5.06	70.2 ± 13.39^a^	87.12 ± 7.05^ab^	174.695	<0.001

Results are expressed as mean ± SD. LDH: lactate dehydrogenase; ALT: alanine aminotransferase; AST: aspartate aminotransferase. ^a^Significantly different from the control group. ^b^Significantly different from the type 1 diabetic patients. *p* < 0.05 is considered significant.

**Table 7:** Correlations of the clinical characteristics and diabetic biomarkers with lactate dehydrogenase and inflammatory and coagulation biomarkers in T1DM patients with and without COVID-19 (*N* = 100).

Parameters	LDH (U/l)	CRP (mg/l)	ESR (mm/h)	Ferritin (ng/ml)	D-dimer (*μ*g/ml)
Comorbidities	0.064	0.241^∗^	0.210^∗^	0.077	0.208^∗^
Hydroxychloroquine administration	-0.201^∗^	-0.329^∗∗∗^	-0.370^∗∗∗^	-0.369^∗∗∗^	-0.379^∗∗∗^
FBG (mmol/l)	0.207^∗^	0.451^∗∗∗^	0.534^∗∗∗^	0.563^∗∗∗^	0.540^∗∗∗^
HbA1c (%)	0.172	0.577^∗∗∗^	0.555^∗∗∗^	0.547^∗∗∗^	0.516^∗∗∗^

Results are correlation coefficients (*r*). FBG: fasting blood glucose; HbA1c: glycated hemoglobin; LDH: lactate dehydrogenase; CRP: C-reactive protein; ESR: erythrocyte sedimentation rate; T1DM: type 1 diabetes mellitus. ^∗^*p* < 0.05, ^∗∗∗^*p* < 0.001.

## Data Availability

The data used to support the findings of this study are available from the corresponding author upon request.
